# Movement-related cortical potentials in paraplegic patients: abnormal patterns and considerations for BCI-rehabilitation

**DOI:** 10.3389/fneng.2014.00035

**Published:** 2014-08-27

**Authors:** Ren Xu, Ning Jiang, Aleksandra Vuckovic, Muhammad Hasan, Natalie Mrachacz-Kersting, David Allan, Matthew Fraser, Bahman Nasseroleslami, Bernie Conway, Kim Dremstrup, Dario Farina

**Affiliations:** ^1^Department of Neurorehabilitation Engineering, University Medical Center GoettingenGoettingen, Germany; ^2^Institute of Computer Science, Georg-August UniversityGoettingen, Germany; ^3^School of Engineering, University of GlasgowGlasgow, UK; ^4^Department of Biomedical Engineering, NED University of Engineering and TechnologyKarachi, Pakistan; ^5^Center for Sensory-Motor Interaction, Aalborg UniversityAalborg, Denmark; ^6^Queen Elizabeth National Spinal Injury Unit, Southern General HospitalGlasgow, UK; ^7^Department of Biomedical Engineering, University of StrathclydeGlasgow, UK; ^8^Academic Unit of Neurology, Trinity College DublinDublin, Ireland

**Keywords:** EEG, movement related cortical potentials, spinal cord injury, central neuropathic pain, BCI

## Abstract

Non-invasive EEG-based Brain-Computer Interfaces (BCI) can be promising for the motor neuro-rehabilitation of paraplegic patients. However, this shall require detailed knowledge of the abnormalities in the EEG signatures of paraplegic patients. The association of abnormalities in different subgroups of patients and their relation to the sensorimotor integration are relevant for the design, implementation and use of BCI systems in patient populations. This study explores the patterns of abnormalities of movement related cortical potentials (MRCP) during motor imagery tasks of feet and right hand in patients with paraplegia (including the subgroups with/without central neuropathic pain (CNP) and complete/incomplete injury patients) and the level of distinctiveness of abnormalities in these groups using pattern classification. The most notable observed abnormalities were the amplified execution negativity and its slower rebound in the patient group. The potential underlying mechanisms behind these changes and other minor dissimilarities in patients’ subgroups, as well as the relevance to BCI applications, are discussed. The findings are of interest from a neurological perspective as well as for BCI-assisted neuro-rehabilitation and therapy.

## Introduction

Movement-related cortical potentials (MRCP) reflect brain electrical activity related to the execution of overt or covert movements. MRCP resulting from either imagery or attempt of motor volition are often investigated in a cue-based paradigm (MacKay and Bonnet, 1990; Ulrich et al., 1998). In paired cue-based paradigms for Brain-Computer Interfaces (BCI), the user is asked to prepare for a movement following the first cue and to execute the movement following the second cue. The readiness potential, which is the leading part of the MRCP and precedes the movement execution, may be movement specific (Shibasaki et al., 1980) when there is only one movement option, or may present general preparation for an action (Walter et al., 1964) when there are several choices for movements. Following the execution cue, the MRCP comprises components known as premotor positivity (Deecke et al., 1976; Castro et al., 2013), motor potential (Deecke et al., 1969) and reafferent potential (Bötzel et al., 1997) related to the kinesthetic feedback once the movement has occurred.

MRCPs are influenced by impairments of the sensory-motor system. Castro et al. (2013) compared MRCPs in three subject groups: healthy individuals who executed movement of the left and right leg, healthy subjects who only prepared for the same movements, and chronic complete spinal cord injured (SCI) patients who imagined the same movements. They observed that the amplitudes of the readiness potential and motor potential were lower in SCI patients than in healthy subject executing the movement, but were comparable between SCI patients and healthy participants who only prepared for the movements. All SCI patients had a complete injury with no preserved sensation under the level of the injury, thus in that study it was not possible to distinguish the effects of sensory and motor loss, specifically complete/incomplete injury in the sensory pathways. Therefore, our first research question is related to the role of sensory information, which can be investigated in complete/incomplete SCI subgroups.

A frequently overlooked co-morbidity of paralysis is Central Neuropathic Pain (CNP), present in 40% of the SCI population, equally affecting paraplegic and tetraplegic patients with complete or incomplete injury (Siddall et al., 2003). CNP is a consequence of an injury to the somato-sensory system (Haanpää et al., 2011), and as such it originates at the cortical level. Functional magnetic resonance imaging (fMRI) studies showed that this type of pain modulates the activity of the motor cortex (Gustin et al., 2010) of both paralyzed “painful” limbs and non-paralyzed limbs. In a recent study, Vuckovic et al. (2014) compared event-related synchronization/desynchronization (ERD/ERS; Pfurtscheller and Lopes Da Silva, 1999) in patients with paraplegia and CNP, patients with paraplegia and no pain and healthy individuals with no pain. Patients with CNP had strongest ERD in the theta, alpha and beta bands, while ERD was less expressed in healthy participants. Patients with no pain (PNP) had the weakest ERD. However, it is not clear if the presence of CNP would equally affect MRCP and ERD/ERS, as it is believed that the two signal modalities have different origins (Babiloni et al., 1999; Pfurtscheller and Lopes Da Silva, 1999). This raises our second research question: the role of pain in MRCPs of SCI patients, which can be studied in pain/no-pain SCI subgroups.

The role of abnormality patterns in MRCP is relevant in BCI-rehabilitation applications. Recently a BCI system based on MRCP was proposed and tested on healthy subjects and stroke patients (Niazi et al., 2011, 2013; Xu et al., 2014a,b). The MRCP was used in this system as a trigger signal (brain switch) to control an external device, such as function electrical stimulation (FES) or an active orthosis. This paradigm was shown to promote activity-dependent cortical plasticity in healthy subjects (Mrachacz-Kersting et al., 2012; Niazi et al., 2012; Xu et al., 2014b) and stroke patients (Mrachacz-Kersting et al., 2013). SCI patients with incomplete injury are ideal candidates for a combined sensory-motor therapy, as the one proposed by using the MRCP as brain switch. However, the characteristics of MRCPs in these patients are not known yet and this information is relevant for the design of a detector based on MRCP waveforms. Hence, the implication on potential applications of BCI for SCI patients is the third research question we will address.

This study presents the initial step in developing an MRCP-based BCI system for SCI patients. For this purpose, we investigated the difference in MRCP morphology between SCI patients and healthy subjects, as well as the unique features of MRCP in sub-groups of patients with different degrees of CNP and scale of impairment (complete or incomplete paralysis). Further, the related issues for BCI rehabilitation are discussed.

## Methods

### Subjects

Eight healthy volunteers (HV) and 14 SCI patients, with either complete or incomplete paralysis, participated in this study. The neurological level of injury was determined using the American Spinal Injury Association (ASIA) Impairment Classification (Marino et al., 2003). SCI patients were further classified on the basis of presence or absence of CNP, below the level of the injury. Inclusion criteria for patients with pain (PWP) was that they were at least 1 year post-injury, were treated for CNP for at least 6 months, had a pain level ≥5 on the Visual Numerical Scale (VNS) and had the injury at level T1 or lower. Inclusion criteria for PNP were that they were at least 1 year post-injury, with injury at level T1 or lower. General exclusion criteria for all three groups were age under 18 or over 55, existence of any other chronic or acute pain at the time of the experiment, brain injury or other known brain condition that would influence EEG interpretation or would prevent the patients from understanding the experimental task. Details about the subjects’ self-reported information are presented in Table 1.

**Table 1:** **Patient information**.

Nr	Level of injury	ASIA*	Time after injury	Pain VNS^‡^	Years with pain
1	T5	A	7	7	7
2	L1	B	15	7	15
3	T6/T7	D	4	7	3
4	T6/7	B	25	10	24
5	T6	B	N/A	N/A	N/A
6	T8	B	11	9	11
7	T12	B	33	6	4
8	T7	A	7	/	/
9	T7	B	7	/	/
10	L1	A	6	/	/
11	T2	A	2	/	/
12	T5	B	15	/	/
13	T4	A	9	/	/
14	T7	A	15	/	/

* ASIA stands for the American Spinal Injury Association, whose impairment classification was used to determine the neurological level of injury.

*^‡^ VNS is Visual Numerical Scale, which was used for pain level assessment*.

Informed consent was obtained from all participants, and the study protocol was approved by the University of Strathclyde Ethical Committee for the HV group and the National Health Service for Greater Glasgow and Clyde Ethical Committee for the SCI group.

### Experiment protocol

**Figure 1:**
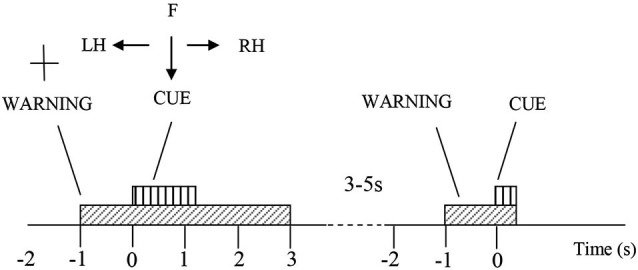
**Experiment protocol**. At *t* = −1 a cross appears on the screen, indicating the participant should prepare for a motor imagery. One second later, an arrow pointing to right, left or down, and the participant were asked to perform a motor imagery of their right hand (RH), left hand (LH) or feet (F) accordingly, till the cross disappear at *t* = 3 s. Afterwards there is a rest period of 3–5 s, before the next trial begins.

The participants were comfortably seated at a desk, at a distance of approximately 1.5 m from the computer screen on which visual instructions were provided. They were instructed to look at the center of the screen and to perform motor imagery following visual cues while minimizing eye movements. For each trial, at *t* = −1 s a readiness cue (a cross +) appeared and would remain for 4 s (Figure 1). At *t* = 0 s an initiation cue, presented as an arrow, appeared next to the cross sign, for a duration of 1.25 s. The arrow pointed either to the left, right or down, corresponding to the motor imagery tasks of left hand waving (LH), right hand waving (RH) and tapping with both feet (F), respectively. The participants were asked to continue to perform imaginary movements until the cross disappeared from the screen (3 s after the initiation cue appeared). In total, 60 trials of each of the three type of motor imagery were performed by each subject in one session. The trials were divided in groups of 10 for each type of imaginary movement (LH, RH and F). Sequences of motor imagery tasks appeared in a random order and at random 3–5 s intervals.

We instructed the participants to perform motor imagery, and we asked them specifically to imagine, not to attempt movements. However, it should be noted that while HV practiced motor imagery, SCI patients might have also attempted to move their paralyzed limb.

A 61-channel EEG recording was performed with an EEG device (Synamps^2^, Neuroscan, USA). The electrodes were placed according to the standard 10-10 locations. EOG was recorded from three channels around the right eye. EEG and EOG were recorded with respect to the linked ear reference and the ground was at AFz. In addition, EMGs were recorded from the right and the left wrist extensor muscles and right foot dorsiflexor using the bipolar inputs to the Synamps device. The purpose of EMG recording was to check for the absence of any voluntary movements when the subjects attempted motor imagery. The sampling frequency was 1000 Hz. The electrode impedance was kept below 5 kΩ during all measurements.

### Signal pre-processing

The EEG signal was down-sampled to 250 Hz and pre-processed with a band-pass filter at 0.1–3 Hz (second order butter-worth), followed by the large Laplacian filter of the respective central channels (Cz and C3 for foot and right hand, respectively) and eight second-nearest channels around them (Fz, FC1, FC2, C3, C4, CP1, CP2, and Pz for foot, and F3, FC5, FC1, T7, Cz, CP5, CP1, and P3 for right hand, respectively). This was done to reject the common mode noise and thus increase the signal-to-noise ratio (McFarland et al., 1997; Niazi et al., 2011). For each trial, the segments between *t* = −2 s and *t* = 6 s, with respect to the cue onset, were extracted as MRCP. All trials were visually inspected to reject trials that were potentially corrupted by artifacts and noise. All trials were of good quality and no trial needs to be rejected.

### Statistical analysis of MRCP morphology across subject groups

We performed the following three pair-wise analyses:

1. **HV vs. SCI**: healthy volunteers vs. all SCI patients independent of the level of injury or the presence of pain (8 HV vs. 14 SCI);
2. **PNP vs. PWP**: SCI patients with no pain vs. SCI patients with CNP (7 PNP vs. 7 PWP). Both PNP and PWP group contained patients with complete and incomplete injury;
3. **CP vs. IP**: SCI patients with complete injury (ASIA A complete loss of motor and sensory functions under the level of the injury) vs. SCI patients with incomplete injury (ASIA B/C/D with some sensations preserved under the level of the injury) (6 CP vs. 8 IP). Both CP and IP groups contained patients with and without pain.

In order to compare MRCP morphology between groups, a Wilcoxon rank-sum test was utilized for statistical analysis. Three comparisons were performed for each type of movement imagery. The null hypothesis was that, for each type of movement within each group, the MRCPs have the same average value at the same temporal location. For each comparison, the entire 8 s long interval was divided into 0.1 s long segments and statistical analysis was performed between groups separately for the 80 segments of each case. The statistical significance level was set to 0.05, with a Holm-Boniferroni correction (Holm, 1979) applied (smallest *p*-value was 0.05/80).

### Classification of MRCPs

Following the above statistical analysis, a two-class classification was performed on MRCPs of each task (feet and right hand) corresponding to the three pairs defined above, i.e., HV vs. SCI, PNP vs. PWP, and CP vs. IP. The classification was performed with a dimensionality reduction algorithm called locality persevering projection (LPP; He and Niyogi, 2003; He et al., 2005), followed by a k-nearest-neighborhood (kNN) classifier. LPP, a manifold-based method, was demonstrated to be superior than linear methods such as PCA and LDA when data have clear nonlinear characteristics (He et al., 2005). LPP can preserve the data structure in the original manifold when projecting data into lower linear feature space, of which classic linear dimensional reduction algorithm such as PCA or SVD is not capable. It was previously used for MRCP detection, in which it outperformed linear match filter method (Xu et al., 2014a). A five-fold cross-validation was used to validate the classification accuracy. The classifier was trained with randomly selected 4/5 of single-trial MRCPs and the remaining 1/5 were considered as testing sets. The LPP algorithm was used to project the training samples into a lower dimensional space, while preserving its intrinsic structure in its original manifold, as in Xu et al. (2014a). The reduced dimension was chosen as 60% of the original data dimension, which was proved to be optimal for MRCP detection (Xu et al., 2014a). Next, the projected data in this LPP subspace were used to train the classifier. In the subsequent testing step, testing samples were projected into the LPP sub-space obtained through training, which was then classified using the trained kNN into either class of the pair (e.g., HV or SCI). The classification performance was quantified with the classification accuracy, i.e., the percentage of correctly classified trials with respect to the total number of testing trials.

Aiming at investigating the temporal discriminant information in the MRCPs among different groups, the classification was not performed on the entire MRCP segments, but with processing window of segments at different temporal location as well as with different segment lengths. This process was done by sliding the starting point of the processing window, from *t* = −2 s to *t* = 3 s (step size 0.1 s). At each starting point, the length of the window also changed from 1 s to 3 s (step size 0.1 s). As the movement imagery was performed until *t* = 3 s, it was not practically useful to process signals 3 s after the movement onset.

## Results

### MRCP morphology

**Figure 2:**
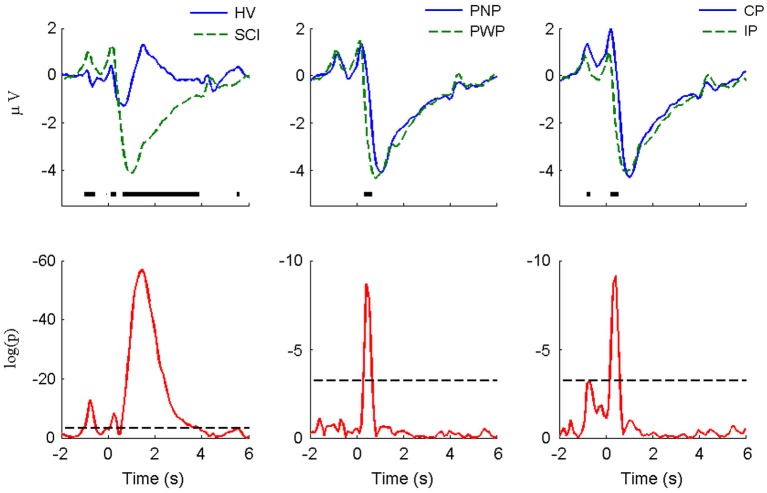
**MRCP morphology and statistical comparison for large Laplacian Cz (motor imagery of foot)**. The figures in the upper row present the average MRCP from different groups (HV vs. SCI, PNP vs. PP, and CP vs. IP). The thick horizontal lines indicate the portions in which statistically significant difference was detected using the Wilcoxon rank-sum test with Holm-Boniferroni correction. The *p*-values of the statistical tests were presented in the lower row, with logarithm scale (log*p*). The dashed horizontal line indicates the minimal significance level in the Holm-Bnifeernoi correction procedure, i.e., log(0.05/80), since there were 80 simultaneous tests in each case.

The MRCPs of foot imagery are compared for the subject groups (i.e., HV vs. SCI, PNP vs. PWP, and CP vs. IP) in Figure 2. The amount of MRCP segments is equal to the product of number of trials and number of subjects. As stated in Section Experiment Protocol, the number of trials is 60 for each type of task by each subject. E.g., we had 8 HV and 14 SCI, the amount of segments for HV and SCI is 480 and 840, respectively. The difference is particularly pronounced for the case of HV vs. SCI. In general, the amplitude of MRCP for the SCI group was significantly greater than that for the HV group (peak-to-peak value: 5.6 ± 6.3 μV vs. 2.7 ± 3.4 μV). The CP group’s MRCP amplitude was also slightly greater than the IP group, while there was only a small difference in amplitude for PNP vs. PWP. The evoked responses following the readiness cue (the “+” sign) and the initiation cue (arrows) are clearly visible in all cases.

#### Period of general preparation for movement

During the period *t* = −1 s to *t* = 0 s, i.e., after the “+” appeared and before the arrow appeared, SCI subjects had a significantly larger positivity than HV subjects. This significant difference lasted until −0.7 s, i.e., 300 ms after the appearance of the “+” sign. This is an indication of altered (enhanced) response to a movement related visual cue from the SCI patients. A significant difference was found for the CP vs. IP group: CP patients had larger visual-motor positivity than IP subjects. This result suggested that complete loss of sensory information (CP group) from the foot enhanced the potential, compare to subjects with some remaining sensory input (IP group). These results imply that the level of deafferentation is positively related to the magnitude of visual evoked potentials. On the other hand, no statistical significance was found in the PWP vs. PNP group, indicating that presence of pain does not affect the magnitude of the preparation potentials.

**Figure 3:**
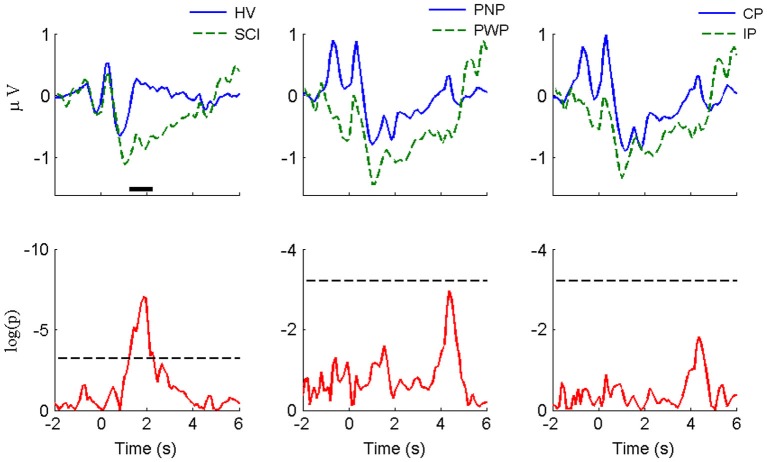
**MRCP morphology and statistical comparison for large Laplacian C_3_ (motor imagery of right hand)**. The convention and legends are the same as in Figure 2.

The visual-motor potential from motor imagery of the hand at C3 during this period (Figure 3) was smaller in magnitude compared with that of foot imagery at Cz. For HV vs. SCI groups, similar to foot imagery, a difference was found around −0.7 s. The difference did not reach statistical significance, probably because it was much smaller both in amplitude and duration than in the case of foot imagery. No statistically significant difference was observed for the CP vs. IP group. Similar to the case of foot imagery, no significant difference was found for PNP vs. PWP group.

A difference at Cz between HV and PWP is because SCI had stronger positivity at around *t* = −0.7 at Cz (painful limbs) than they had at C3 which corresponds to a non-paralyzed limb. It is true that we showed Cz for MI of legs and C3 for MI of right hand, but we also checked that SCI has larger positivity at Cz than HV even for motor imagery of hands. For HV there does not seem to be a difference between Cz and C3 in a period *t* = −1 s to *t* = 0 s.

In summary, during this period of general preparation for movement, the subjects did not know what type of motor imagery should be performed, so the visual-motor potential was not task-specific. Therefore, the consistent results between foot imagery and hand imagery, other than the overall magnitude difference, are expected. This is particularly the case for HV vs. SCI group, where significance was detected at this point for foot imagery. While for hand imagery a distinct peak of the *p*-value existed, no statistical significance could be established.

#### Period of movement specific-preparation and covert motor execution

As presented in Figure 2, during the period of *t* = 0 s to *t* = 3 s of foot motor imagery, a statistically significant difference can be noticed in all three pairs of comparisons. The largest difference was observed between HV and SCI. SCI patients had significantly larger amplitude of the positive peak at 300 ms. The MRCP negativity of the SCI group was also significantly larger than for the HV subjects. The rebound from the negativity of the HV group appeared around *t* = 1 s and then returned back to baseline around *t* = 3 s; for the SCI group, the rebound was more gradual, reaching the baseline at approximately *t* = 6 s without a second positive peak. The main difference between PNP group and PWP group was located between *t* = 0 s and *t* = 1 s, where descending for the PWP group was faster than that for the PNP group. Similarly the largest difference between CP and IP group could be noticed in the first 0.5 s following the directional cue. The CP group presented higher amplitude of the positive peak and faster decreasing slope than IP group.

For hand imagery, the differences after the initialization cue were much smaller than difference for feet imagery for all groups. This is expected since none of the subjects had sensory or motor impairments of the upper extremities. Still, there was a statistically significant difference between HV and SCI groups in part of the rebound phase (from 1.2 s to 2.3 s). However, there is no statistical difference between PNP and PWP group and between CP and IP group.

In summary, largest differences during the period following the directional cue were noticed, as expected, between HV and SCI group and they were present for both paralyzed and non-paralyzed limbs. Smaller differences, both in magnitude and duration, also existed between PWP and PNP group and for CP and IP group, for motor imagery of feet. However no statistically significant difference was observed for motor imagery of right hand in either of the patient sub-groups.

### Classification performance

**Figure 4:**
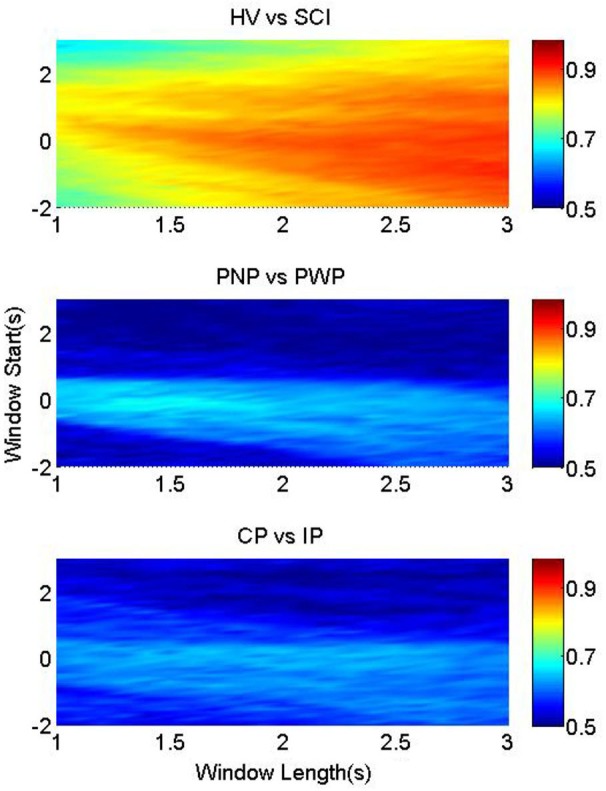
**Classification accuracy for foot imagery**. The color-bars identify the classification accuracies of MRCP segments from three pairwise groups, with onset from *t* = −2 s to *t* = 3 s, and length between 1 s and 3 s. These three figures from upper to bottom represent the results of HV vs. SCI, PNP vs. PWP, and CP vs. IP, respectively.

Figure 4 illustrates the classification accuracies of the three groups, as a function of the starting point and the length of the processing windows. It was possible to classify between foot imagery of the HV and SCI group with higher accuracy than the two patient sub-groups (Figure 4). This is in accordance with the largest statistical difference found between the MRCP of HV vs. SCI, as presented in Figure 2.

The highest average accuracy of HV vs. SCI was 90.5% (at window start = −1 s and window length = 3 s), while those of PNP vs. PWP and CP vs. IP were 68.7% (at window start = −0.1 s and window length = 1.4 s) and 65.1% (at window start = 0.2 s and window length = 2 s), respectively.

In addition, the accuracies changed according to the window start and length, and the patterns of this change are different among the three pairwise groups. For HV vs. SCI, the part with accuracies ~90% was located in the bottom right corner, where the starting point was mostly before *t* = 0 s, and the length was larger than 2.5 s. High classification accuracies (74%) were achieved even when only a 1 s period of general preparation (*t* = −1 s till *t* = 0 s) was used to classify between the two groups. As the analysis time window moved towards the movement specific period, shorter windows were sufficient to achieve high classification accuracy, indicating largest difference between HV and SCI during the period of task specific motor imagery. This high classification performance resulted from the large difference in MRCPs between HV and SCI, as shown in Figure 4.

**Figure 5:**
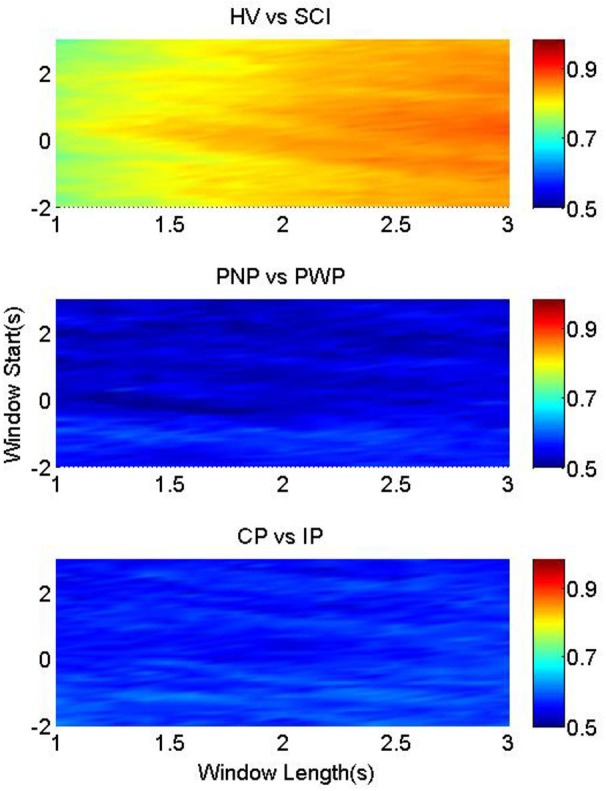
**Classification accuracy for right hand**. The color-bars identify the classification accuracies of MRCP segments from three pairwise groups, with onset from *t* = −2 s to *t* = 3 s, and length between 1 s and 3 s. These three figures from upper to bottom represent the results of HV vs. SCI, PNP vs. PWP, and CP vs. IP, respectively.

Similar observation holds for motor imagery of the right hand (Figure 5), indicating a general influence of paralysis on the signal characteristics. This indicates that paralysis globally changes preparation of movement, not restricted to the paralyzed limb.

Nevertheless, the distributions of accuracies for the other two groups are notably different. For imaginary movement of feet the higher accuracy (>65%) part for PNP vs. PWP were limited in a small strip around window onset *t* = 0 s, with window length of 1.2–1.7 s. This strip with higher classification accuracy exactly matched the MRCP range with lower *p*-values, and higher statistical significance in Figure 2. These results indicate statistically significant difference between these two groups in a period of general preparation and in the period of the early preparation/initiation of the covert movement.

The area with accuracies higher than 60% for CP vs. IP was also small, but the shape was evidently different from that of PNP vs. PWP. It was an approximately horizontal strip where the onset was around *t* = 0 s and the length ranges from 1.5 s to 2.5 s. This shows that preserved sensation do not considerably influence MRCP in the general preparation of movement but it does influence preparation for specific movement of a part of the body with preserved sensation vs. part of the body with no sensation.

The classification rate between PNP and PWP in motor imagery of the right hand could reach 50% only if the period of general preparation was included in the analysis. This indicates that the presence of CNP influences the general preparation of movement in the painful/non-painful and paralyzed/non-paralyzed limb. In a study on the ERS/ERD of the same group of patients (Vuckovic et al., 2014), a generalized influence of pain on movement of painful and non-painful limbs was also found.

For right hand between CP and IP group, classification accuracy was slightly higher for the period of general preparation, but it has no clear pattern anywhere else.

## Discussion

This study presented analysis of the difference in MRCP morphology of covert movement between HV and patients with spinal cord injury, through direct statistical comparison and through pattern classification. This has implications on performance of BCI control systems based on MRCP which has mostly been tested on healthy individuals.

The aim of this study was to compare MRCP between HV and patients during both general and movement specific preparation, therefore a period of general focus (general non-specific movement preparation) was also taken into account.

While HV presented a relatively homogeneous group, the situation of chronic paraplegic patient is more complex. In the current study, we further categorized the SCI patient volunteers into two sub-groups, based on: the severity of paralysis and presence of chronic CNP pain. By combining patients with respect to different criteria, we investigated the influence of loss of motor control (HV vs. SCI), loss of sensation (CP/IP), and presence of CNP.

### Distinction between HV and SCI groups

The largest differences in MRCP morphology were found between healthy and general mixed group of paraplegic patients during covert movements of feet in all phases of MRCP. It is interesting that significant difference was found even during the period of visual stimuli. Presenting a general warning sign produced significant difference between the groups (with a peak around 300–400 ms post-stimuli). This can be explained by the combined visual-motor nature of this potential, especially as the motor area is heavily involved in their generation but do not show much sensitivity to motor task parameters at this specific positive peak (Ulrich et al., 1998). It may be speculated that this positivity is generated by the increased firing rate of cortical neurons in motor areas, as in similar instruction delay experiments on primates that showed comparable delays after the cues (Cisek and Kalaska, 2004). Higher amplitude of the peak in SCI patients might be possibly related to higher effort/concentration in SCI patients expecting to imagine/attempt movement of a paralyzed limb. In SCI patients a motor potential in period *t* > 0 s had significantly higher negative peak with a rebound potential also called reafferent potential (Castro et al., 2013)- being delayed for several seconds. The amplitude of the rebound potential was also much lower in SCI group, which is explained by its relation to the kinesthetic feedback.

Statistically significant differences in MRCP morphology between these two groups were also found for motor imagery of the right hand, over electrode location C3, though to a smaller extent. This demonstrated the global influence of paralysis on modified EEG responses, and is in accordance with previous studies looking into either spontaneous (Tran et al., 2004; Boord et al., 2008; Vuckovic et al., 2014) or evoked brain activity (Vuckovic et al., 2014) in SCI patients. While the larger negativity during imagination of movement in paraplegic patients resembled the study by Lacourse (1999), there were many detailed differences that may originate from different cue type or EEG referencing.

### Distinction between SCI subgroups

The analyzed group of patients was mixed with respect to the severity of paralysis and presence of chronic pain, therefore the results could not be conclusive. Therefore we further compared MRCP in patients with and without CNP. CNP is known to affects the activity of the motor cortex (Vuckovic et al., 2014), thus potentially influencing the morphology of MRCPs. Assuming that CNP is unrelated to the completeness of injury, patients with complete and incomplete injury were mixed. Analysis showed much smaller difference between patients with and without pain than between healthy and general SCI population.

#### The effect of CNP

It is known that CNP equally affects patients with complete and incomplete SCI (Siddall et al., 2003). A previous EEG study by Vuckovic et al. (2014), performed on the same group of volunteers, and the same experimental paradigm, demonstrated a difference in brain response between SCI patients with and without CNP, as well as between both groups of SCI patients and able-bodied volunteers. Those differences were wide spread over the sensory-motor cortex and were not restricted to imagination of paralyzed, “painful” part of the body. The study was based on ERD/ERS and was primarily interested in a time period after presentation of the directional cue, in a period *t* = 0.4–2 s.

The MRCP results in the current study are therefore not in accordance with the ERD/ERS analysis on the same patient group (Vuckovic et al., 2014). While paralysis resulted in reduced ERD, presence of CNP increased ERD. Therefore that study showed larger difference in cortical response between patients with and with no CNP than between patients with CNP and healthy subjects. The differences were pronounced within the first 2 s after presentation of the directional cue, while in the current study, the difference in MRCP morphology was significant in a short interval (0.3–0.6 s). This supports the idea of different origin of ERD and MRCP, which has been reported in the literature. The source of MRCP is related to the cerebellar-thalamus-cortical pathway (Babiloni et al., 1999; Rektor et al., 2001), while ERD is related to the thalamo-cortical feedback loops (Pfurtscheller and Lopes Da Silva, 1999). Since CNP is known to be not related to cerebellum activities (Vuckovic et al., 2014), the difference in the neurophysiological origin of MRCP and ERD supports the observed difference of MRCP and EDR with respect to the presence/absence of CNP.

#### The effect of the completeness of injury

Finally patients’ MRCP were compared on the basis of the completeness of the injury, assuming that presence of CNP does not have a large effect on MRCP. For MRCP measured over Cz for motor imagery of feet, the largest difference was found in periods of both general preparation and covert movement execution in patients with complete injury.

Castro et al. (2013) compared MRCP in chronic paraplegic patients with complete injury and in healthy subjects during covert movement execution of left or right leg. Although in that study larger MRCP could be noticed over electrode locations C3, C4 and Cz, no difference was found when MRCP was averaged over all electrodes. In the current study, we analyzed only electrode location where we expected largest MRCP. We also preprocessed the signal using large Laplacian filter that might have additionally enhances MRCP over these areas.

A general conclusion is that while both CNP and presence/absence of sensation affect the morphology of MRCP in paralyzed limb, the factor that most strongly influencs the MRCP is the lack of motor control, resulting in large difference between healthy subjects and general SCI group.

### Implications for BCI-rehabilitation

Results of MRCP classification supported the morphological analysis. In general the highest classification accuracy was found in the time windows which corresponded to the time windows of statistically significant difference between the groups. While classification accuracy between able-bodied group and patients exceeded 90%, classification between different patients groups was not higher than 65%. This further supports the idea that for MRCP-based BCI systems, paralysis is a factor that needs to be considered as it has a strong influence on the MRCP morphology. Therefore, the following issues should be seriously taken into consideration when developing MRCP-based BCI, especially cue-based BCI, for SCI patients.

Firstly, although the larger magnitude might probably improve the BCI performance in SCI patients, the prolonged rebound should be treated carefully with a long interval between trials. On the other hand, SCI PNP have weaker ERD than the able-bodied volunteers (Vuckovic et al., 2014), resulting in reduced BCI classification accuracy (Pfurtscheller et al., 2009). This implies that for SCI patients, BCI systems which relay on MRCP might have better classification accuracy, with greater consistency among patients.

Further, the lack of statistical difference of patient sub-groups with the distinct peaks in the corresponding *p*-value curves (lower panels of Figure 2) probably resulted from a much larger variability of MRCP in patients (both within and between subjects). This would affect the performance of BCI system for these patients.

Although almost no significant difference was found in the MRCP morphology between PWP and with no pain, chronic SCI patients with CNP might experience worsening of pain during prolonged MI practice (Gustin et al., 2010).

Finally, while it was the motor impairment (compared to the remaining sensory function or presence of pain) that had a considerable effect on the MRCP waveforms and can affect BCI performance, the clinical practice and therapy is by no means independent from these factors.

### Limitations

The healthy group, which was comparable to the size of SCI subgroups, was not large. As the magnitude of MRCPs for hand was smaller than that of the foot task (see Figure 3), it would be better to have more subjects to increase statistical power of the analysis, so that statistical significance might be revealed in some cases where no significance was detected in the current analysis. Nevertheless, there were 60 trials for each type of task by each subject, so we had hundreds of segments (e.g., 480 for HV and 840 for SCI) for statistical comparison. In fact, we did find significance for HV vs. SCI for hand motor imagery (see Figure 3), but not for SCI subgroups. Given the very large *p*-value for PNP vs. PWP and CP vs. IP (only one peak is close to significance level), we believe the likelihood of missing potential significant differences was not large.

Other factors, besides the abnormal patterns in MRCP, could also contribute to the BCI design. One of these factors that was not discussed in this study is volitional inhibition (Logan, 1994), which refers to the cortical involvement of suppression of on-going voluntary movements. Even though previous studies found that volitional inhibition activates motor cortexes (Coxon et al., 2006; Mirabella et al., 2011; Mattia et al., 2012), it does not attract much attention from the majority of BCI research community (Mirabella, 2012). Recently, Ifft et al. (2012) attempted to decode the volitional inhibition from brain signal, but there is still more work leaving for BCI researcher to dig information from overt movement as well as the volitional control (Fetz, 2007).

## Conflict of interest statement

The authors declare that the research was conducted in the absence of any commercial or financial relationships that could be construed as a potential conflict of interest.
